# The Complete Mitochondrial Genome of the Stalk-Eyed Bug *Chauliops fallax* Scott, and the Monophyly of Malcidae (Hemiptera: Heteroptera)

**DOI:** 10.1371/journal.pone.0055381

**Published:** 2013-02-04

**Authors:** Teng Li, Cuiqing Gao, Ying Cui, Qiang Xie, Wenjun Bu

**Affiliations:** Institute of Entomology, College of Life Sciences, Nankai University, Tianjin, China; The University of Hong Kong, China

## Abstract

*Chauliops fallax* Scott, 1874 (Hemiptera: Heteroptera: Malcidae: Chauliopinae) is one of the most destructive insect pests of soybean and rice fields in Asia. Here we sequenced the complete mitochondrial genome of this pest. This genome is 15,739 bp long, with an A+T content of 73.7%, containing 37 typical animal mitochondrial genes and a control region. All genes were arranged in the same order as most of other Heteroptera. A remarkable strand bias was found for all nine protein coding genes (PCGs) encoded by the majority strand were positive AT-skew and negative GC-skew, whereas the reverse were found in the remaining four PCGs encoded by the minority strand and two rRNA genes. The models of secondary structures for the two rRNA genes of sequenced true bugs and Lygaeoidea were predicted. 16S rRNA consisted of six domains (domain III is absent as in other known arthropod mitochondrial genomes) and 45 helices, while three domains and 27 helices for 12S rRNA. The control region consists of five subregions: a microsatellite-like region, a tandem repeats region and other three motifs. The unusual intergenic spacer between *tRNA-H* and ND4 only found in the species of Lygaeoidea, not in other heteropteran species, may be the synapomorphy of this superfamily. Phylogenetic analyses were carried out based on all the 13 PCGs showed that Chauliopinae was the sister group of Malcinae and the monophyly of Lygaeoidea.

## Introduction

The stalk-eyed bug, *Chauliops fallax* Scott, 1874, is an important pest of bean plants such as soybean and a minor cause of pecky rice in China, Japan and Korea [1]–[3]. Ecology and controlling methods of this species were studied in past years [2]. However, no molecular markers have been used to investigate the population genetic structure or evolutionary patterns of *C. fallax*, which might facilitate the managements of this pest.

The genus *Chauliops* was described by Scott (1874) as a lygaeid genus, then it was included in Heterogastrinae [4]. Breddin (1907) raised *Chauliops* to a subfamily Chauliopinae [5]. Later on, Chauliopinae and Malcinae were considered closely allied by many authors based on morphological characters [6]. Štys (1967) gave family status to the Malcidae (only including Chauliopinae and Malcinae) also based on morphological characters [7], and his opinion was accepted by nearly all subsequent authors [8]. Hua *et al.* (2008) provided the mitochondrial genome (mt-genome) data of Malcinae (genus *Malcus*) [9], however, no molecular data have been published in Chauliopinae, and the relationships of Chauliopinae and Malcinae have not been confirmed by molecular evidence so far.

In recent years, the numbers of complete mitochondrial (mt) genome sequences of insects have a rapid increase due to its relative short in length (14–20 kb) and easy to get the whole genome, and widely use in inferring phylogenetic relationships [10]. Up to now, the complete mt-genomes of 282 species of insects, and the complete or nearly complete mt-genomes of 35 species among 30 families of Heteroptera, which includes 76 families totally [11], [12], are available at NCBI (status September 10, 2012). However, the number of sequenced heteropteran mt-genomes is still very limited relative to the species-richness of Heteroptera.

In this study, we describe the complete mitochondrial genome sequence of the *C. fallax* and provide analyses of the nucleotide composition, codon usage, compositional biases, RNA secondary structure, and evaluate the phylogenetic relationship of Chauliopinae and Malcinae in Heteroptera based on the sequences of protein coding genes (PCGs). We compare the conserved sequences of RNA secondary structures of sequenced true bugs and Lygaeoidea species respectively, which may be helpful for aligning rRNA sequences correctly and reconstructing improved phylogeny trees in the future. Moreover, we also discover some potential conserved motifs of RNA secondary structure in Lygaeoidea.

## Results and Discussion

### Genome organization and structure

**Figure 1:**
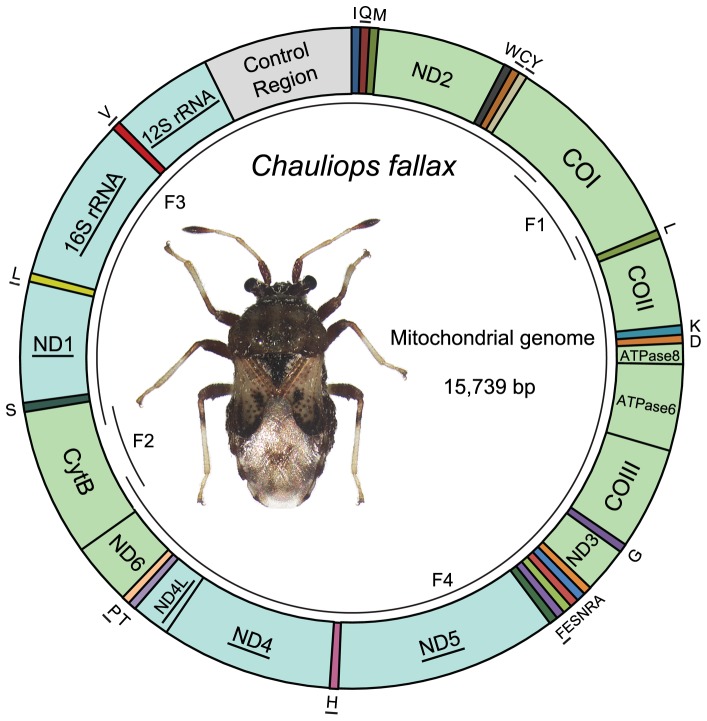
Structure of the mitochondrial genome of *Chauliops fallax* (GenBank accession number JX839706). Gene names without underline indicate the direction of transcription of the majority strand (J-strand), and with underline indicate the minority strand (N-strand). The tRNA genes are denoted by the color blocks and are named using single-letter amino acid abbreviations. Overlapping arcs (F1–F4) within the circle indicated the PCR-amplified fragments.

The complete mitochondrial genome sequence of *C. fallax* was a double-stranded circular DNA molecule of 15,739 bp in size and has been deposited in the GenBank (Accession number: JX839706; Figure 1). This mt-genome totally contained the typical 37 genes (two rRNAs, 13 PCGs and 22 tRNAs) and a large non-coding region (control region), with the same gene order as observed in most other true bugs [13] (Table 1). Gene overlaps were observed at 16 gene junctions and involved a total of 67 bp which may make the genome relatively compact; the longest overlap (16 bp) existed between ND4L and *tRNA-Thr*. The two gene pairs ATP8/ATP6 and ND4L/ND4 overlapped same seven nucleotides (ATGATAA). In addition to the control region, 101 nucleotides were dispersed in eight intergenic spacers, ranging in size from 1 to 59 bp. The longest spacer sequence was located between *tRNA-His* and ND4.

**Table 1:** Organization of the *Chauliops fallax* mitochondrial genome.

Gene	Strand	Position	Anticodon	Size (bp)	Start codon	Stop codon	Intergenic nucleotides^a^
tRNA-Ile	J	1–67	GAT	67			
tRNA-Gln	N	65–133	TTG	69			−3
tRNA-Met	J	133–200	CAT	68			−1
ND2	J	201–1193		993	ATA	TAA	0
tRNA-Trp	J	1192–1256	TCA	65			−2
tRNA-Cys	N	1268–1331	GCA	64			11
tRNA-Tyr	N	1333–1398	GTA	66			1
COI	J	1405–2943		1539	TTG	TAA	6
tRNA-Leu	J	2939−3004	TAA	66			−5
COII	J	3005–3683		679	ATT	T-	0
tRNA-Lys	J	3684–3755	CTT	72			0
tRNA-Asp	J	3755–3819	GTC	65			–1
ATPase8	J	3820–3978		159	ATA	TAA	0
ATPase6	J	3972–4634		663	ATG	TAA	−7
COIII	J	4634–5420		787	ATG	T-	−1
tRNA-Gly	J	5421–5486	TCC	66			0
ND3	J	5487–5840		354	ATA	TAA	0
tRNA-Ala	J	5841–5904	TGC	64			0
tRNA-Arg	J	5906–5975	TCG	70			1
tRNA-Asn	J	5972–6039	GTT	68			−4
tRNA-Ser	J	6039–6108	GCT	70			−1
tRNA-Glu	J	6108–6174	TTC	67			−1
tRNA-Phe	N	6176–6243	GAA	68			1
ND5	N	6243−7946		1704	ATT	TAA	−1
tRNA-His	N	7933–7998	GTG	66			−14
ND4	N	8058–9374		1317	ATG	TAA	59
ND4L	N	9368–9664		297	ATT	TAA	−7
tRNA-Thr	J	9649–9711	TGT	63			−16
tRNA-Pro	N	9712–9774	TGG	63			0
ND6	J	9777–10253		477	ATC	TAA	2
CytB	J	10253–11386		1134	ATG	TAG	−1
tRNA-Ser	J	11385–11454	TGA	70			−2
ND1	N	11475–12398		924	ATT	TAA	20
tRNA-Leu	N	12399–12464	TAG	66			0
16s rRNA	N	12465–13729		1265			0
tRNA-Val	N	13730–13797	TAC	68			0
12S rRNA	N	13798–14591		794			0
Control		14592–15739		1148			0

a Numbers correspond to nucleotides separating a gene from an upstream one; negative numbers indicate that adjacent genes overlap.

### Transfer RNAs

All of the 22 typical animal tRNA genes were found in *C. fallax* mt-genome, ranging from 63 to 72 bp. Most of the tRNAs could be folded into the classic cloverleaf secondary structure except for *tRNA-Ser (GCT)*, in which its dihydrouridine (DHU) stem simply formed a loop (see Figure S1). The amino acid acceptor stem (7 bp) and the anticodon loop (7 nt) had extremely low variability, and the most variable in size was the stems and loops of DHU and TΨC, which the loop size (3–10 bp) was more variable than the stem size (2–5 bp). The length of the anticodon stems was conservative, with the exception of *tRNA-Ser (GCT)* which possessed a long optimal base pairing (9 bp in contrast to the normal 5 bp) and a bulged nucleotide in the middle for the AC stem.

A total of 28 unmatched base pairs exist in the *C. fallax* mitochondrial tRNA secondary structures, 26 of which were G-U pairs located in the amino acid acceptor stem (8 bp), the DHU stem (10 bp), the anticodon stem (4 bp), the TΨC stem (4 bp), and the remaining two U–U mismatches in the amino acid acceptor stem of *tRNA-Ala* and *tRNA-Leu (TAG)* respectively. Additionally, 24 mismatches were significantly biased in eight tRNA genes which were encoded on the minority strand (N-strand), and the others were found on the majority strand (J-strand).

### Ribosomal RNAs

**Figure 2:**
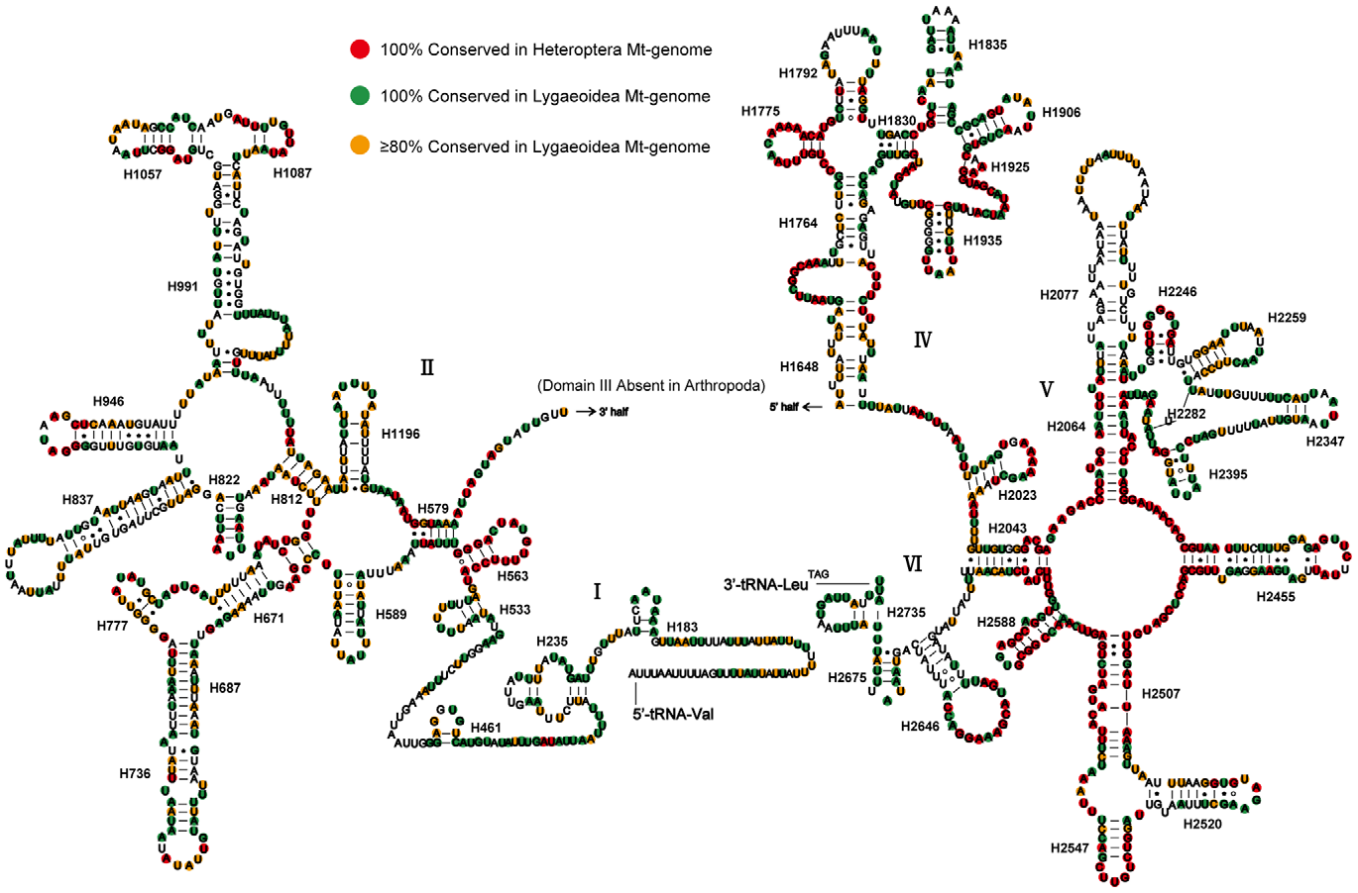
Predicted secondary structure of the mitochondrial 16S rRNA of *Chauliops fallax*. The 100% conserved sites among sequenced true bugs were plotted with red background. The 100% and more than 80% conserved sites among sequenced Lygaeoidea species were plotted with green and orange background, respectively. Canonical Watson-Crick interactions are represented by a dash, non-canonical guanine-uracil interactions are represented by an asterisk, and all other non-canonical interactions are represented by a hollow circle. Roman numerals denote the conserved domain structure.

**Figure 3:**
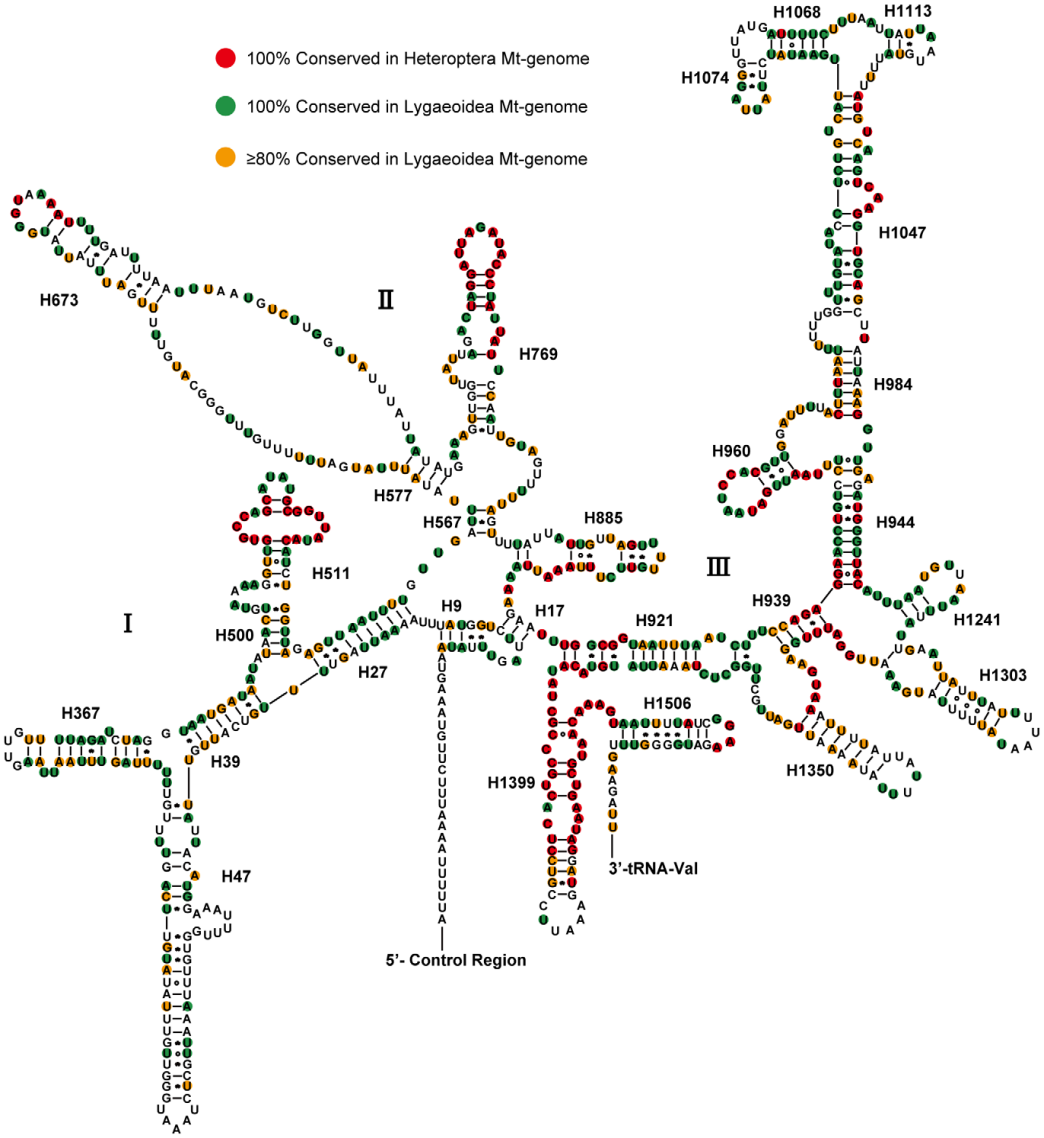
Predicted secondary structure of the mitochondrial 12S rRNA of *Chauliops fallax*. The annotation is the same as for Figure 2.

The ends of *C. fallax* rRNA genes were assumed to extend to the boundaries of flanking genes, because it was impossible to precisely determined by DNA sequencing alone [14]. The 16S rRNA (large rRNA subunits) was assumed to fill up the blanks between *tRNA-Val* and *tRNA-Leu (TAG)*. The 12S rRNA (small rRNA subunits) was located between *tRNA-Val* and the non-coding region. 16S rRNA has a length of 1,265 bp with an A+T content of 77.6%, while 12S rRNA has a length of 794 bp with an A+T content of 74.6%. The secondary structure of *C. fallax* 16S rRNA consisted of six structural domains (domain III is absent as in other arthropod mt-genomes) and 45 helices (Figure 2). The secondary structure of 12S rRNA consisted of three structural domains and 27 helices (Figure 3).

In 16S rRNA, domains IV and V are more conserved than domains I, II, and VI by the alignment of the sequenced species in Heteroptera and also in Lygaeoidea. Some helices (H563, H1775, H2064, H2507, and H2588) are highly conserved in both sequence and secondary structure among most heteropteran mtDNA, and only few nucleotides differences are found either in the terminal loops or in the terminal couplets of helices. In domain II, a conserved helix could not be observed within the available heteropteran mtDNA, but the helix H579, H822 and the internal loop of helix H991 are highly conserved in Lygaeoidea mtDNA. Helix H837 forms a long stem structure with a small loop in the terminal as frequently found in other insects [15]. In domain IV, the initial 5 bp of helix H1792 form hydrogen bonds as in most insects [16], and the first nucleotide pair in this helix often forms a UU interaction which has been observed in some helices of the insect 12S rRNA gene [17]. Although the terminal half of H1792 may pair as in many insects, these interactions are less conserved [16]. Accordingly, we leave the terminal half of H1792 unpaired in *C. fallax*. The secondary structure of the helix H1835 is similar to that proposed for *Drosophila melanogaster* [15], *Apis mellifera* [18] and *Ruspolia dubia* [19], which are different with the structure of some true bugs proposed by Li *et al*. [20]. In domain V, most helices are highly conserved in secondary structure, with the exception of H2077 and H2347. In terms of secondary structure and alignment, helix H2077 is the most problematic region with no apparent conserved motifs [16]. The helix H2347 is greatly variable among many insects mtDNA, and in *C. fallax* this region consisting of just the terminal 3 paired bases, which is similar to that proposed for *Zygaena sarpedon lusitanica* [21]. In addition, some helices (H183, H736, H991, H1057, H1087, H1196, H1648, H2077, H2347 and H2735) are greatly variable in both sequence and secondary structure, and the sequences from H183 to *tRNA-Val* are most different among most insect mtDNA.

In 12S rRNA, the sequence and secondary structure of domain III is more conserved than the other two parts (domains I and II) among most heteropterans. The 5′ end of the 12S rRNA was made up of a long, unpaired sequence followed by a pseudoknot formed by 5 bp stem H9 and the 5′ portion of stem H17. The helix H27 is probably ten base pairs long in *C. fallax*, while the secondary structure assumed identical with the fruit fly model (for *Drosophila melanogaster*) [15] and the Gutell model (for *Apis mellifera*) [18]. However, helix H27 was 8 bp long in some other models [22], [23] for the reasons of two additional base pairings at the distal end of the helix is unclear. The helix H47 is highly variable among heteropterans and difficult to align with few nucleotides which only conserved in Lygaeoidea. A consistent secondary structure for this region could not be found even within all the available mitochondrial 12S rRNA structures. The possible folds of this section presumed for *C. fallax* consists a long stem, an internal loop and a short terminal loop, which was predicted by the Mfold web server [24]. From helices H567 to H769, the secondary structure of this circle section is highly variable among the studied taxa and only aligned ambiguously. An exception is the distal section of helix H769 is extremely conserved as in other insects [22]. In domain III, helices from H921 to H960 are highly conserved among Lygaeoidea. However, the most complicated portion of 12S rRNA located in the stem H1047 and the associated stems H1068, H1074 and H1113, possibly because its high AT bias and several non-canonical base pairs across many other insects [25]. Due to the evidence found for helix H1068 in insects [26], a six base-pair-long stem mostly comprising 5′-GAAUAU-3′ on one side and 5′-AUUUUC-3′ on the other. Helix H1303 consists of a lone nucleotide pair at the base of the helix, an internal bulge, and a distal stem containing three UU base pairs. Helix H1399 is more conserved than any other helices of 12S rRNA across true bugs, but the terminal loop is highly variable both in length and sequence.

### Protein coding genes

Twelve of the 13 PCGs of *C. fallax* initiated with ATN as start codon (four with ATG, four with ATT, three with ATA and one with ATC) (Table 1). The only exception was the COI gene, which used TTG as a start codon. This non-traditional start codon for the COI gene was also observed in other true bugs [13], and dipterans [27], [28]. Most PCGs stopped with the complete termination codon: ten with TAA (ND2, COI, ATP8, ATP6, ND3, ND5, ND4, ND4L, ND6 and ND1) and one with TAG (CytB). The remaining two (COII and COIII) were terminated with a single T adjacent to a downstream tRNA gene on the same strand. The phenomenon that single T acts as termination codon was common in insect mt-genomes and it had been presumed that the complete termination codon TAA could be generated by posttranscriptional polyadenylation [29].

### Nucleotide composition and codon usage

For the whole mt-genome of *C. fallax*, the nucleotide composition was significantly biased toward A and T. The A+T content was 73.7% (A = 44.8%, T = 28.9%, C = 16.7%, G = 9.6%), which is a common value among known hexapod mt-genomes ranging from 62.4% in *Atelura formicaria* (Zygentoma) [30] to 87.4% in *Diadegma semiclausum* (Hymenoptera) [31]. The average A+T content of all PCGs, tRNAs, rRNAs and the control region is 72.9%, 77.2%, 76.4% and 72.4%. The lowest A+T content is 67.5% in COI, while the highest is 81.8% in ATP8 (Table 2). The nucleotide skew statistics [32] of all PCGs show that the J-strand PCGs (AT-skew = 0.11, GC-skew = −0.22) were much less TA- and GC-skewed than the N-strand PCGs (AT-skew = −0.40, GC-skew = 0.32), and the N-strand tRNAs had also higher GC-skewed than the J-strand tRNAs. This kind of strand bias of nucleotides composition has been generally related to asymmetric mutational constraints in the process of replication [33].

**Table 2:** Nucleotide composition of the *Chauliops fallax* mitochondrial genome.

Feature	Length (bp)	A%	C%	G%	T%	A+T%	AT-skew	GC-skew
Whole genome	15739	44.8	16.7	9.6	28.9	73.7	0.22	−0.27
Protein coding genes	10992	33.0	13.9	13.3	39.8	72.8	−0.09	−0.03
First codon position	3664	36.8	12.4	18.4	32.4	69.2	0.06	0.20
Second codon position	3664	20.6	18.7	14.1	46.6	67.2	−0.39	−0.14
Third codon position	3664	41.6	10.7	7.3	40.4	82.0	0.01	−0.19
Protein coding genes-J	6762	39.5	17.4	11.2	32.0	71.5	0.11	−0.22
First codon position	2254	42.5	15.1	17.6	24.8	67.3	0.26	0.08
Second codon position	2254	21.6	21.0	13.3	44.1	65.7	−0.34	−0.23
Third codon position	2254	54.4	16.0	2.7	27.0	81.4	0.34	−0.71
Protein coding genes-N	4230	22.5	8.5	16.6	52.4	74.9	−0.40	0.32
First codon position	1410	27.6	8.1	19.7	44.6	72.2	−0.24	0.42
Second codon position	1410	18.9	15.0	15.4	50.7	69.6	−0.46	0.01
Third codon position	1410	21.1	2.3	14.7	62.0	83.0	−0.49	0.73
tRNA genes	1463	39.4	9.6	13.2	37.8	77.2	0.02	0.16
tRNA genes-J	933	42.0	11.3	11.6	35.2	77.2	0.09	0.01
tRNA genes-N	530	34.7	6.8	16.0	42.5	77.2	−0.10	0.41
rRNA genes	2059	29.2	7.7	15.9	47.2	76.4	−0.24	0.35
Control region	1148	46.3	19.8	7.8	26.1	72.4	0.28	−0.43
ATP6	663	41.6	17.7	8.3	32.4	74.1	0.12	−0.36
ATP8	159	56.0	13.8	4.4	25.8	81.8	0.37	−0.52
COI	1539	35.6	17.5	15.1	31.8	67.5	0.06	−0.07
COII	679	41.7	17.7	11.1	29.6	71.3	0.17	−0.23
COIII	787	39.3	17.8	12.3	30.6	69.9	0.12	−0.18
CytB	1134	35.8	19.2	12.4	32.5	68.3	0.05	−0.21
ND1	924	20.1	8.7	18.4	52.8	72.9	−0.45	0.36
ND2	993	42.0	14.6	9.0	34.4	76.4	0.10	−0.24
ND3	354	42.4	21.8	7.9	28.0	70.3	0.20	−0.47
ND4	1317	26.1	9.0	14.7	50.2	76.3	−0.32	0.24
ND4L	297	26.9	6.4	14.1	52.5	79.5	−0.32	0.38
ND5	1704	20.5	8.3	17.4	53.8	74.4	−0.45	0.35
ND6	477	43.4	13.8	6.7	36.1	79.5	0.09	−0.35
16s rRNA	1265	30.0	7.4	15.0	47.6	77.6	−0.23	0.34
12s rRNA	794	28.0	8.1	17.4	46.6	74.6	−0.25	0.37

**Figure 4:**
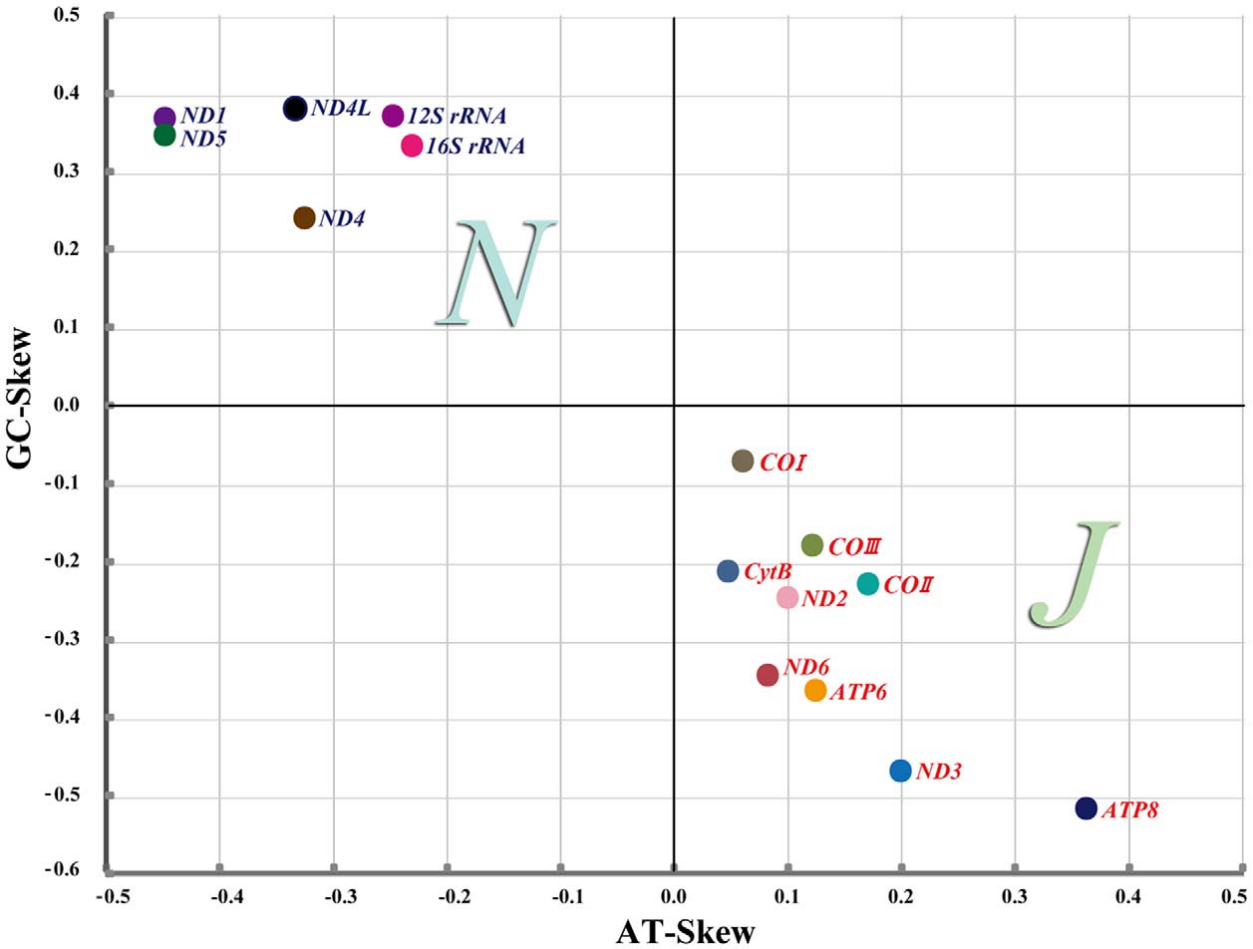
AT- and GC-skews of *Chauliops fallax* mitochondrial genome. 13 protein coding genes (PCGs) and 2 rRNAs are represented in different color circles. Letter J means the majority strand (J-strand), N means the minority strand (N-strand).

Besides, in *C. fallax*, it was interesting that each of the PCGs of J-strand was positive AT-skew and negative GC-skew, whereas the reverse was observed in each of the PCGs of N-strand (Figure 4). This remarkable phenomenon has not been reported for any insect mt-genome before. Unfortunately, the mechanism of this phenomenon is unclear. However, there were reports that the value of GC skew was associated with replication orientation and AT skew varies with gene direction, replication and codon positions [34]. To deeply understand the mechanism of this phenomenon, more research work about mt-genomes sequences and function are needed to be done.

**Figure 5:**
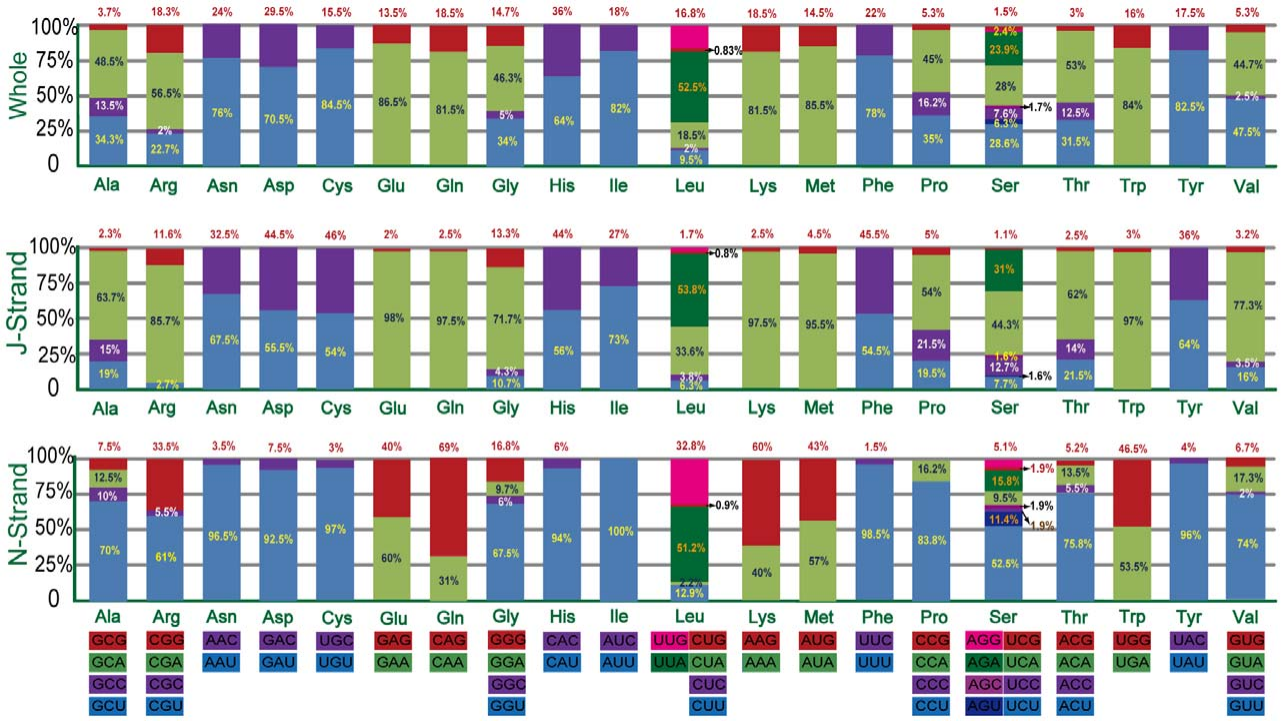
Percentage of synonymous codon usage of each amino acid in the *Chauliops fallax* mitochondrial genome. Codon families are provided on the x-axis.

The nucleotide bias toward AT was also reflected in the codon usage (Table 2). The analysis of the base composition at each codon position of 13 PCGs showed that the third codon position (82%) was higher in A+T content than the first (69.2%) and second (67.2%) codon positions. The mt-genome of *C. fallax* contained 3,664 codons totally, while 2,254 codons (61.5%) were found on the J-strand and 1,410 codons (38.5%) on the N-strand. Over all, four most prevalent codons in *C. fallax*, Ile (ATT) (8.68%), Met (ATA) (7.97%), Phe (TTT) (6.87%) and Leu (TTA) (6.65%) were all composed wholly of A and/or T, which may play an important role for the whole mt-genome high A+T content. In addition, the most infrequently used codons were NNG (267 codons, 7.3%) and the most frequently used codons were NNA (1,523 codons, 41.6%). The fourfold degenerate codon usage presented a strong bias towards adenine (A) at the third codon of J-strand PCGs whereas uridine (U) shows preponderance on the N-strand, except the Ser (AGN) whose most frequently used codons are ended with A (Figure 5). The twofold degenerate codon usage demonstrated definite bias favoring A/U over G/C at the third codon position on both strands, except the Gln (CAR) and Lys (AAR) of the N-strand favoring G rather than A. All codons are present on both strands of *C. fallax* mtDNA PCGs, but AGG and CGC codons are not observed in the J-strand, and CUC, AUC, CCC, CCG and CGA codons in the N-strand, reflecting the influence of a strong biased codon usage [35].

### Non-coding regions

The mt-genome of *C. fallax* includes three major non-coding regions of more than 20 bp: spacer 1 was 59 bp between *tRNA-His* and ND4, spacer 2 was 20 bp between *tRNA-Ser* and ND1, and spacer 3 was 1,148 bp with 72.4% A+T content between 12S rRNA and *tRNA-Ile* (I)- *tRNA-Gln* (Q)- *tRNA-Met* (M) gene cluster (Figure 1).

Spacer 1 is a feature common to each of the five Lygaeoidea mt-genomes (38 bp in Berytidae, 40 bp in Malcinae, 59 bp in Chauliopinae, 72 bp in Colobathristidae, and 124 bp in Geocoridae) which have been sequenced to date but is not found in other heteropterans. Additionally, in Malcinae, it has been reported that one subregion of the intergenic spacer between *tRNA-His* and ND4 has an exactly repeated counterpart in the control region (34 nt, Blast E-value: 2e-15), and thought it may be the autapomorphy of Malcidae [9]. However, in Chauliopinae, the sister group of Malcinae, no copy of this spacer was found across all the mt-genome. Hence, the repetition may be the autapomorphy only for Malcinae, not including the subfamily Chauliopinae.

**Figure 6:**
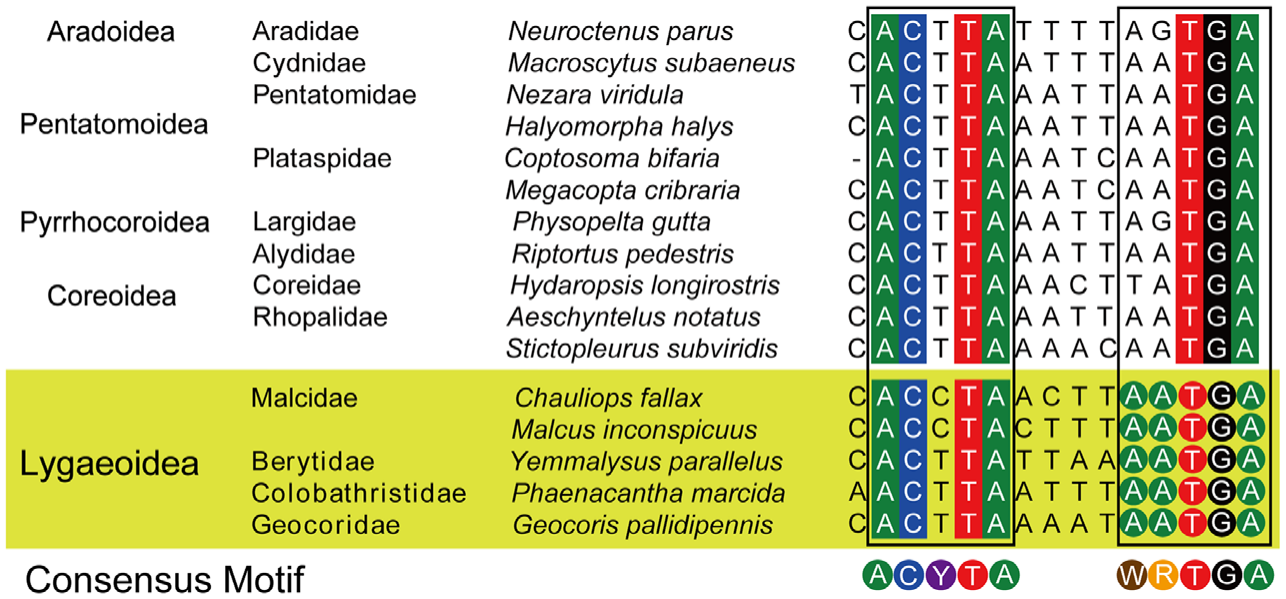
Alignments of the spacers between *tRNA-Ser (TGA)* and ND1 across Lygaeoidea and other Pentatomomorpha species. The alignments were generated by plotting the identities in different colors, and a gap as a dash.

Spacer 2 is common to most insect mt-genomes. Among these spacers, there are two consensus motifs in a conserved sequence block (CSB) region (Figure 6), which may indicate that they undergo a common intermediate stage of tandem duplication and random loss (TDRL) process [36]. There is a 5 bp motif, AATGA, which is conserved across the members of Lygaeoidea, and to a lesser extent across the infraorder Pentatomomorpha, WRTGA. The another 5 bp motif, ACTTA, which is conserved across the members of Pentatomomorpha with the exception of Malcidae (Malcinae + Chauliopinae), ACCTA, which may be the autapomorphy of Malcidae and provide another evidence for the monophyly of Malcidae.

**Figure 7:**
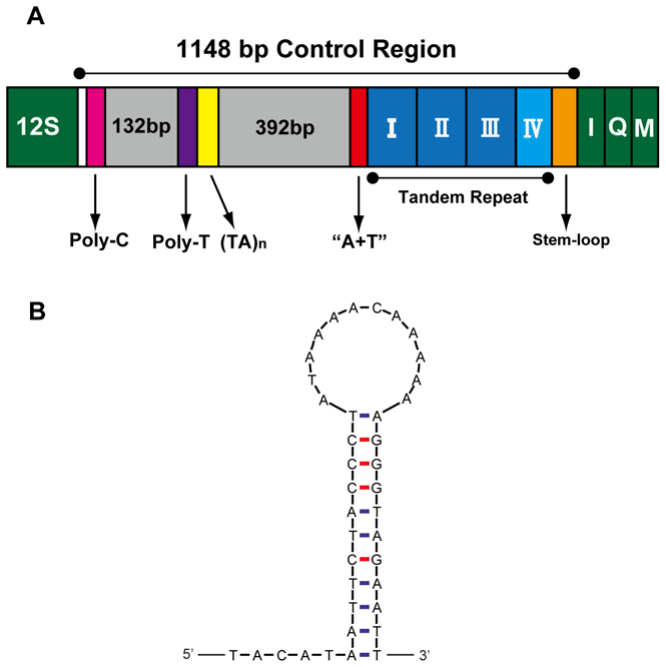
Control region of *Chauliops fallax* mitochondrial genome. (A) Structure elements found in the control region of *C. fallax*. The control region flanking genes 12S rRNA, *tRNA-I, tRNA-Q*, and *tRNA-M* are represented in green boxes; “(TA)n” (yellow) indicates the microsatellite-like region; “A+T” (red) indicates high A+T content region; the blue and azury boxes with roman numerals indicate the tandem repeat region; orange boxes represent the stem-loop region. (B) The putative stem-loop structure found in the control region.

Spacer region 3 is considered as the control region identified in other mt-genomes [37] which includes the origin sites for transcription and replication [38]. In some arthropods mt-genomes, the control region was reported to have one to four of these four different motifs: tandem repeats, poly-thymine (poly-T) sequence, a subregion of even higher AT richness, and a stem-loop structure [39]. The control region of *C. fallax* contained all these four motifs and could be divided into five parts (Figure 7A): (1) at the 5′-end of the control region is a 7 bp poly-C structure, which was also found in other insects [20], [25]; (2) a 8 bp poly-T stretch and a microsatellite-like region ((TA)_4_ (GATATA)_2_); (3) a 35 bp region heavily biased toward A+T (91.4%); (4) a 460 bp region contained four tandem repeats including three (I–III) 122 bp repeat units and a partial copy of the repeat (IV) 94 bp, which were identified by tandem repeats finder server [40]; (5) a potential stem-loop secondary structure was found at the end of control region, however, without ‘TATA’ sequence existed at the 5′ end and ‘G(A)nT’ at the 3′ end (Figure 7B). In the second region, the poly-T stretch may play a role in the control of transcriptional or may be the site of replication initiation [41]. The microsatellite-like region, located 188 bp from 12S RNA, was rare and only been reported in *Stenopirates* sp. [20] among all studied heteropterans. In the fourth region, tandem repeats are common in the control region for most insects, and length variations may be caused by a variable copy number of repetitive elements, which produces obvious size variation in the entire mt-genome [42]. The existence of tandem repeats can be explained by replication slippage mechanism [42], [43].

### Phylogenetic relationships

**Figure 8:**
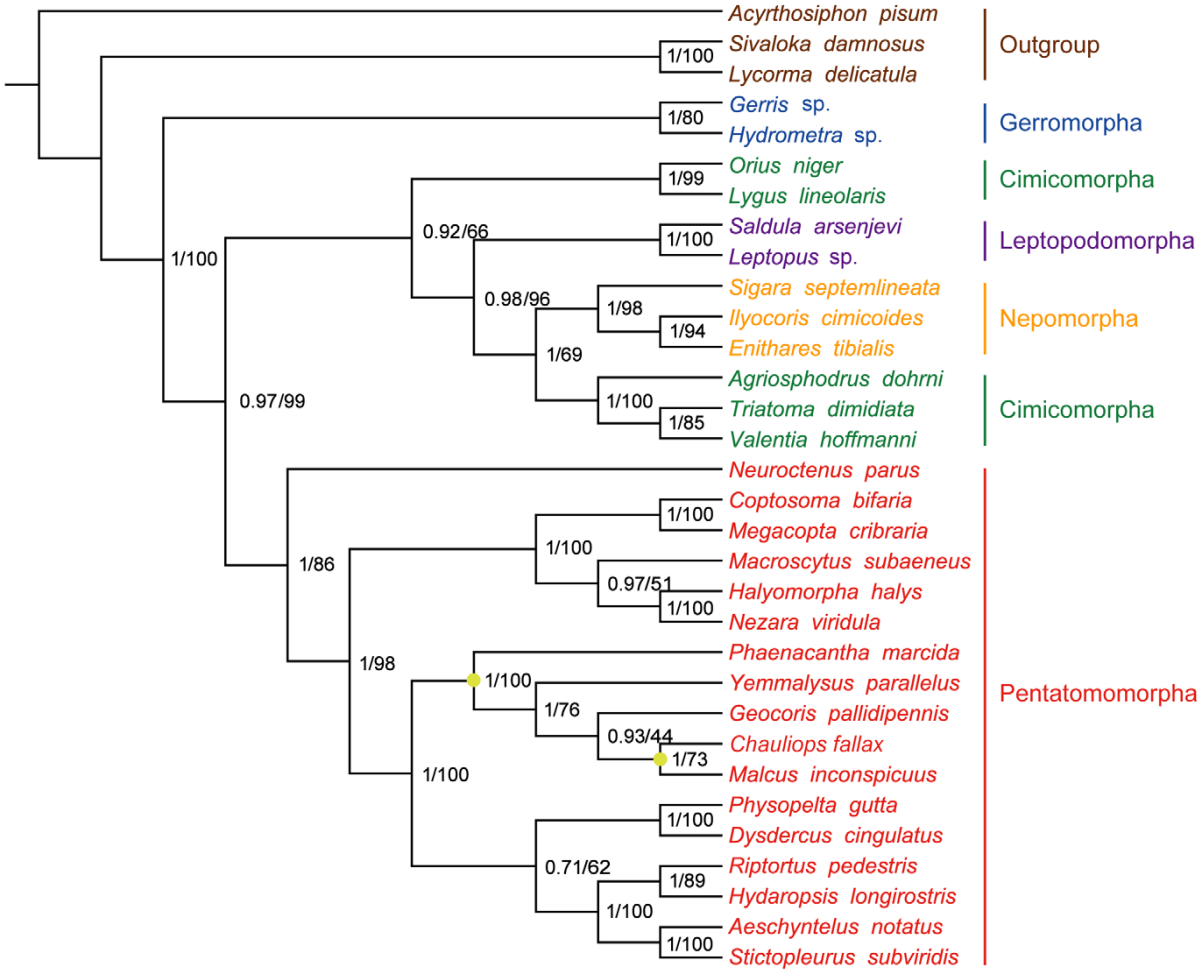
Phylogenetic tree inferred from the sequences of 13 PCGs of 32 hemipteran species. Numbers at the nodes are Bayesian posterior probabilities (left) and ML bootstrap values (right). Two yellowish dots on the tree indicate the clades of Lygaeoidea and Malcidae, respectively.

Phylogenetic analysis was performed with the large data set, 29 heteropteran species as ingroups and other 3 hemipterans as outgroups (*Acyrthosiphon pisum* [44], *Sivaloka damnosus* [45] and *Lycorma delicatula* [13]). Bayesian inference and ML analyses recovered fully bifurcating trees with the same topology (Figure 8). In the present study, the topology of infraordinal relationships of Heteroptera is similar to previous work [25]. Two Gerromorpha superfamilies were monophyletic in the basal position of these five infraorders. Within Cimicomorpha, Reduviidae was paraphyletic with respect to Anthocoridae and Miridae. In Pentatomomorpha, our results support that Aradoidea and the Trichophora are sister groups as indicated in Xie *et al*. [46]. In Eutrichophora, our study was (Lygaeoidea + (Pyrrhocoroidea + Coreoidea)) but poorly supported by ML and Bayesian inferences, while more extensive taxa sampling was needed in further analysis. In Lygaeoidea, our conclusion was (Colobathristidae + (Berytidae + (Geocoridae + Malcidae))), and the sister-relationship of Malcinae and Chauliopinae was confirmed.

## Materials and Methods

### Ethics statement

No specific permits were required for the insect collected for this study in Zhejiang Province, China. The insect specimens were collected in the soybean field by net sweeping. The field studies did not involve endangered or protected species. The species in the genus of *Chauliops* are common small insects and are not included in the “List of Protected Animals in China”.

### Specimen collection

Adult specimens of *Chauliops fallax* were collected from Denggan Village (29°16.904N, 120°21.189E), Dongyang City, Zhejiang Province, China, on July 1st, 2000. Voucher specimens are deposited in the Insect Molecular Systematic Lab, Institute of Entomology, College of Life Sciences, Nankai University, Tianjin, China. All specimens were preserved in 100% ethanol in field. After being transported to the laboratory, they were stored at −20°C until used for DNA extraction.

### PCR amplification and sequencing

Total genomic DNA was extracted from muscle tissue of thorax by a CTAB-based method [47]. The entire mt-genome of *Chauliops fallax* was amplified in four overlapping PCR fragments by PCR amplification. The primer were modified from previous work [48], and designed from the sequenced fragments (see Table S1).

PCRs were performed with TaKaRa LA Taq under the following conditions: 1 min initial denaturation at 94°C, followed by 30 cycles of 20 s at 94°C, 1 min at 50°C, and 2–8 min at 68°C, and a final elongation for 10 min at 72°C. The PCR products were electrophoresed in 1% agarose gel, purified, and then sequenced by ABI 3730XL capillary sequencer with the BigDye Terminator Sequencing Kit (Applied Bio Systems). All fragments were sequenced with primer walking on both strands.

### Sequence analysis and annotation

Sequence files were proof read and assembled into contigs in BioEdit version 7.0.5.2 [49]. Protein coding regions were identified by ORF Finder implemented by the NCBI website (http://www.ncbi.nlm.nih.gov/gorf/gorf.html) with invertebrate mitochondrial genetic codes. To ensure the accurate boundaries of different genes, protein coding regions and ribosomal RNA genes were compared with published insect mitochondrial sequences with CLUSTAL X version 1.83 [50]. Transfer RNA analysis was conducted using tRNAscan-SE version 1.21 [51] with the invertebrate mitochondrial codon predictors and a cove score cut off of 5. Only a few of tRNA genes that could not be detected by tRNAscan-SE were identified by comparing to other heteropterans. Nucleotide composition and codon usage were analyzed with MEGA 5.0 [52]. Strand asymmetry was calculated using the formulas: AT skew =  [A−T]/[A+T] and GC skew =  [G−C]/[G+C] [32]. The putative control region was inferred using the Mfold web server (http://mfold.rna.albany.edu/) [24] with default settings to identify the regions of potential inverted repeats or palindromes. The tandem repeats of the control region were identified by tandem repeats finder server (http://tandem.bu.edu/trf/trf.html) [40].

### Secondary structure of rRNAs prediction

Both 16S rRNA and 12S rRNA were derived from the secondary structure models proposed for other insects, *Drosophila melanogaster* (Diptera: Drosophilidae) [15], *Apis mellifera* (Hymenoptera: Apidae) [18], *Manduca sexta* (Lepidoptera: Sphingidae) [23], *Ruspolia dubia* (Orthoptera: Conocephalidae) [19] and *Stenopirates* sp. (Hemiptera: Enicocephalidae) [20]. Stem-loops were named according to the convention of Gillespie *et al.* [18] and Cameron *et al.* [23]. The regions lacking significant homology were folded using RNAstructure 5.2 [53] and Mfold web server [24].

### Phylogenetic analyses

Phylogenetic analysis was carried out based on the 29 complete or nearly complete mt-genomes of true bugs from GenBank. Three species from Sternorrhyncha and Auchenorrhyncha were selected as outgroups (see Table S2). DNA alignment was inferred from the amino acid alignment of 13 PCGs using MUSCLE as implemented in the MEGA version 5.0 [52]. Alignments of individual genes were then concatenated to be the data set used to reconstruct the phylogeny excluding the stop codon and the third codon. GPU MrBayes [54] and PHYML online web server [55] were employed to reconstruct the phylogenetic trees with the GTR+I+G model estimated by Modeltest Version 3.7 [56]. In Bayesian inference, two simultaneous runs of 5,000,000 generations were conducted for the matrix. Each set was sampled every 100 generations with a burnin of 25%. Trees inferred prior to stationarity were discarded as burnin, and the remaining trees were used to construct a 50% majority-rule consensus tree. In ML analysis, the parameters were estimated during analysis and the node support values were assessed by bootstrap resampling (BP) calculated using 100 replicates.

## Supporting Information

Figure S1 **Putative secondary structure of the 22 tRNAs identified in the mitochondrial genome of** ***Chauliops fallax*** **.** The tRNAs are labeled with the abbreviations of their corresponding amino acids. Dashes indicate Watson-Crick base pairing and asterisks indicate G-U base pairing.
(TIF)

Table S1 **Primers designed for** ***Chauliops fallax*** **in this study.**
(DOC)

Table S2 **Summary of sample information used in present study.**
(DOC)
