# Supplementary figures and images for: The Complete Mitochondrial Genome of the Stalk-Eyed Bug Chauliops fallax Scott, and the Monophyly of Malcidae (Hemiptera: Heteroptera)
Source: PLoS One. 2013 Feb 4;8(2):e55381. doi: 10.1371/journal.pone.0055381 (PMC3563593; doi:10.1371/journal.pone.0055381)

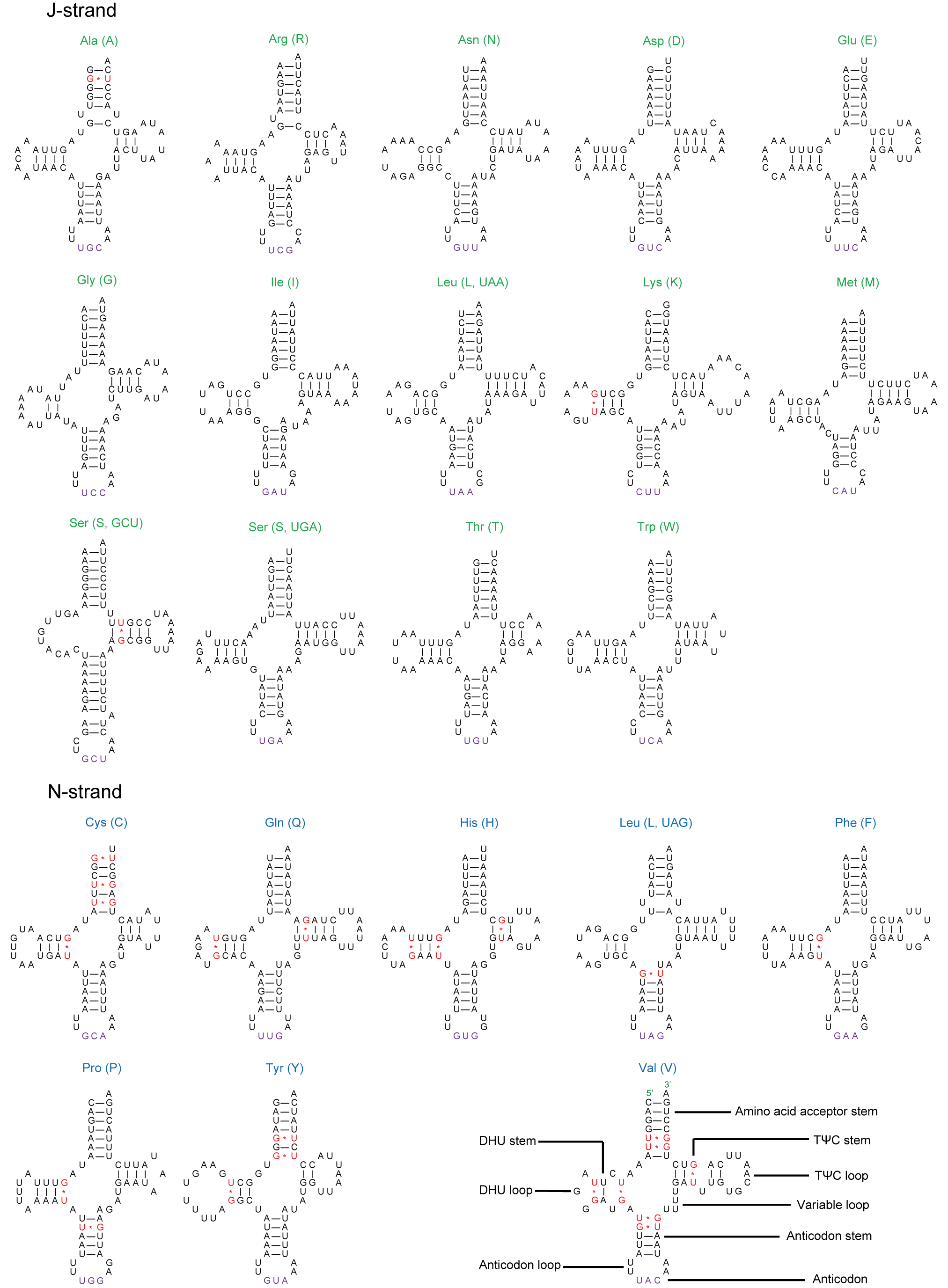

Supplement: Figure S1 — Putative secondary structure of the 22 tRNAs identified in the mitochondrial genome of Chauliops fallax . The tRNAs are labeled with the abbreviations of their corresponding amino acids. Dashes indicate Watson-Crick base pairing and asterisks indicate G-U base pairing. (TIF) [file pone.0055381.s001.tif]
