# Supplementary material for: The Complete Mitochondrial Genome of the Stalk-Eyed Bug Chauliops fallax Scott, and the Monophyly of Malcidae (Hemiptera: Heteroptera)
Source: PLoS One. 2013 Feb 4;8(2):e55381. doi: 10.1371/journal.pone.0055381 (PMC3563593; doi:10.1371/journal.pone.0055381)
Supplement: Table S1 — Primers designed for Chauliops fallax in this study. (DOC) [file pone.0055381.s002.doc]

Table S1. Primers designed for *Chauliops fallax* in this study.

| **Primer** | **Sequence (5’-3’)** |
| --- | --- |
| CO1F | GGAACAGGATGAACAGTTTACCCTCC |
| CO1R | TCTGAATATCGTCGAGGTATTCC |
| CytbF | TATGTTCTTCCCTGAGGACAAATATC |
| CytbR | ATTACACCTCCTAATTTATTAGGAAT |
| CF1F | CGGAGGAGGAGATCCTATTTTATATCAGCTT |
| CF1R | TAGGTGGGCTTTCTAATGGCAAATACGC |
| CF2F | ATAACATGAATTGGTGCCAACCCAGATG |
| CF2R | GCTCCTAGAATAGAGGAAATTCCAGCTAGT |
