# Supplementary material for: The Complete Mitochondrial Genome of the Stalk-Eyed Bug Chauliops fallax Scott, and the Monophyly of Malcidae (Hemiptera: Heteroptera)
Source: PLoS One. 2013 Feb 4;8(2):e55381. doi: 10.1371/journal.pone.0055381 (PMC3563593; doi:10.1371/journal.pone.0055381)
Supplement: Table S2 — Summary of sample information used in present study. (DOC) [file pone.0055381.s003.doc]

Table S2. Summary of sample information used in present study.

| **Order/suborder** | **Infraorder/superfamily** | **Family** | **Species** | **Accession number** |
| --- | --- | --- | --- | --- |
| **Sternorrhyncha** | Aphidoidea | Aphididae | *Acyrthosiphon pisum* | NC_011594 |
| **Auchenorrhyncha** | Fulgoroidea | Fulgoridae | *Lycorma delicatula* | NC_012835 |
|  |  | Issidae | *Sivaloka damnosus* | NC_014286 |
| **Heteroptera** | **Gerromorpha** |  |  |  |
|  | Hydrometroidea | Hydrometridae | *Hydrometra* sp. | NC_012842 |
|  | Gerroidea | Gerridae | *Gerris* sp. | NC_012841 |
|  | **Nepomorpha** |  |  |  |
|  | Corixoidea | Corixidae | *Sigara septemlineata* | FJ456941 |
|  | Notonectoidea | Notonectidae | *Enithares tibialis* | NC_012819 |
|  | Naucoroidea | Naucoridae | *Ilyocoris cimicoides* | NC_012845 |
|  | **Leptopodomorpha** |  |  |  |
|  | Saldoidea | Saldidae | *Saldula arsenjevi* | NC_012463 |
|  | Leptopodoidea | Leptopodidae | *Leptopus* sp. | FJ456946 |
|  | **Cimicomorpha** |  |  |  |
|  | Cimicoidea | Anthocoridae | *Orius niger* | NC_012429 |
|  | Reduvioidea | Reduviidae | *Triatoma dimidiata* | NC_002609 |
|  |  |  | *Valentia hoffmanni* | NC_012823 |
|  |  |  | *Agriosphodrus dohrni* | HM071001 |
|  | Miroidea | Miridae | *Lygus lineolaris* | EU401991 |
|  | **Pentatomomorpha** |  |  |  |
|  | Aradoidea | Aradidae | *Neuroctenus parus* | NC_012459 |
|  | Pentatomoidea | Pentatomidae | *Nezara viridula* | NC_011755 |
|  |  |  | *Halyomorpha halys* | NC_013272 |
|  |  | Cydnidae | *Macroscytus subaeneus* | NC_012457 |
|  |  | Plataspidae | *Coptosoma bifaria* | NC_012449 |
|  |  |  | *Megacopta cribraria* | NC_015342 |
|  | Lygaeoidea | Berytidae | *Yemmalysus parallelus* | NC_012464 |
|  |  | Colobathristidae | *Phaenacantha marcida* | NC_012460 |
|  |  | Malcidae | *Malcus inconspicuus* | NC_012458 |
|  |  |  | *Chauliops fallax* | JX839706 |
|  |  | Geocoridae | *Geocoris pallidipennis* | NC_012424 |
|  | Pyrrhocoroidea | Largidae | *Physopelta gutta* | NC_012432 |
|  |  | Pyrrhocoridae | *Dysdercus cingulatus* | NC_012421 |
|  | Coreoidea | Alydidae | *Riptortus pedestris* | NC_012462 |
|  |  | Coreidae | *Hydaropsis longirostris* | NC_012456 |
|  |  | Rhopalidae | *Aeschyntelus notatus* | NC_012446 |
|  |  |  | *Stictopleurus subviridis* | NC_012888 |
